# Strain Localization in Thin Films of Bi(Fe,Mn)O_3_ Due to the Formation of Stepped Mn^4+^-Rich Antiphase Boundaries

**DOI:** 10.1186/s11671-015-1116-8

**Published:** 2015-10-17

**Authors:** I MacLaren, B Sala, S M L Andersson, T J Pennycook, J Xiong, Q X Jia, E-M Choi, J L MacManus-Driscoll

**Affiliations:** SUPA School of Physics and Astronomy, University of Glasgow, Glasgow, G12 8QQ UK; SuperSTEM Laboratory, STFC Daresbury Laboratories, Keckwick Lane, Warrington, WA4 4AD UK; Department of Materials, University of Oxford, Parks Road, Oxford, OX1 3PH UK; Center for Integrated Nanotechnologies, Los Alamos National Laboratory, Los Alamos, NM 87545 USA; State Key Lab of Electronic Thin Films and Integrated Devices, University of Electronic Science and Technology of China, NO.4, Section 2, North Jianshe Road, Chengdu, 610054 China; Department of Materials Science, University of Cambridge, 27 Charles Babbage Road, Cambridge, CB3 0FS UK; Present Address: Physics of Nanostructured Materials, Faculty of Physics, University of Vienna, Boltzmanngasse 5, A-1090 Vienna, Austria

**Keywords:** Bismuth ferrite, Scanning transmission electron microscopy (STEM), Strain, Thin films, Multiferroic, Antiphase boundaries

## Abstract

The atomic structure and chemistry of thin films of Bi(Fe,Mn)O_3_ (BFMO) films with a target composition of Bi_2_FeMnO_6_ on SrTiO_3_ are studied using scanning transmission electron microscopy imaging and electron energy loss spectroscopy. It is shown that Mn^4+^-rich antiphase boundaries are locally nucleated right at the film substrate and then form stepped structures that are approximately pyramidal in three dimensions. These have the effect of confining the material below the pyramids in a highly strained state with an out-of-plane lattice parameter close to 4.1 Å. Outside the area enclosed by the antiphase boundaries, the out-of-plane lattice parameter is much closer to bulk values for BFMO. This suggests that to improve the crystallographic perfection of the films whilst retaining the strain state through as much of the film as possible, ways need to be found to prevent nucleation of the antiphase boundaries. Since the antiphase boundaries seem to form from the interaction of Mn with the Ti in the substrate, one route to perform this would be to grow a thin buffer layer of pure BiFeO_3_ on the SrTiO_3_ substrate to minimise any Mn-Ti interactions.

## Background

There has been considerable interest in bismuth ferrite due to the fact that it supports simultaneous permanent magnetic [1] and ferroelectric [2] orderings, has potential for use as a multiferroic with strong magnetoelectric couplings [3, 4], and is a rare low bandgap ferroelectric, which is tunable across the whole visible spectrum by B-site doping, and hence could be used as an absorber material with enhanced carrier extraction [5, 6]. Nevertheless, it has proved difficult to create the desired properties in pure BiFeO_3_, and attention has turned to using compositional modification [7–10] and/or substrate-induced strain [11, 12] to alter the structure and properties of the material. Recently, Choi et al. [13] reported the growth of ferroelectric and ferromagnetic films of nominal composition of BiFe_0.5_Mn_0.5_O_3_ on SrTiO_3_ and showed that very careful slow growth was critical to achieving this result. In this work, we report a careful atomic resolution investigation of antiphase boundaries forming close to the film-substrate interface and show, using a careful study with atomic resolution scanning transmission electron microscopy, that even in such high-quality films, there are additional, hitherto, unexpected complexities driven by a tendency to some chemical segregation. These result in the formation of additional antiphase boundaries that have the effect of releasing the compressive elastic strain from the epitaxial growth on the substrate above the boundaries.

## Methods

Films were grown by pulsed laser deposition onto a SrTiO_3_ (STO) substrate held at 640 °C from a Bi(Fe,Mn)O_3_ (BFMO) ceramic target of composition Bi_2_FeMnO_6_ using a KrF laser pulsed at 2–5 Hz and an oxygen pressure of 100 mTorr, as described in more detail in our previous publication [13]. Samples were prepared for scanning transmission electron microscopy using a modified focused ion beam liftout procedure. Firstly, the sample was coated with about 30 nm of carbon; the samples were then prepared using a standard focused ion beam (FIB) liftout procedure, with the orientation of the slice chosen to ensure that the section had a primitive {001} plane of the perovskite in the sample plane. After liftout, the sample was attached onto the side of an Omniprobe copper mount using platinum deposition and then thinned to ~100 nm using 5-kV Ga ions. Final thinning was then performed using a Gatan PIPS equipped with the low-energy upgrade, and the Ar ions were directed onto the sample using angles of +8° and −8° with single-sided sector milling engaged and the orientation of the sample set to ensure that no copper was re-deposited from the support onto the lifted out slice. This final thinning was performed at a low voltage of 500 V for about half an hour.

Scanning transmission electron microscopy was performed using two microscopes. High-angle annular dark-field (HAADF) imaging was performed using a JEOL ARM200F with a cold field emission source and using an accelerating voltage of 200 kV, a probe of semi-convergence angle of 29 mrad and an effective collection angle range for the HAADF detector of about 90–150 mrad. Images were collected at short exposure times of about 10 μs/pixel using a mode of repeated scanning to record 10–30 frames of the same structure for post-processing.

Electron energy loss spectroscopy-spectrum imaging (EELS-SI) was performed using a NION UltraSTEM operated at 100 kV and a Gatan Enfina EELS with a probe angle of 32 mrad and a collection angle of 37 mrad.

Initial processing for quantitative analysis of atom positions was performed by sorting out a number of images from the image stack that are all free of obvious glitches and distortions. These were then aligned using the SDSD plug-in for Digital Micrograph [14] and summed to create a high signal to noise, drift-free image, as done in our previous work [15–18]. Quantitative determination of the atomic positions in the thin film was performed using an experimental Image Analysis plug-in for Gatan Digital Micrograph (courtesy of Dr Bernhard Schaffer, Gatan GmbH), which automatically detects the peaks and fits them using a 2D Gaussian function and then provides a list of peak positions and fit parameters. Further processing, evaluation and plotting of the peak list were then performed using conventional spreadsheets, detailed in previous publications [15–18].

EELS-SI datasets were processed by the following procedure. The datasets were first processed to remove any extraneous X-ray spikes resulting from X-ray generation from stray scattering in the spectrometer. Multivariate statistical analysis was then performed using the plug-in of Lucas et al*.* [19] to separate the real elemental edges from the random noise. The quantification was then performed using an EELS modelling approach [20–23] included in an experimental Digital Micrograph plug-in [24]; since the low-loss datasets were not available, only relative quantification of atomic ratios was performed and the spectra were fitted from 400 to 760 eV to cover the Ti-L_2,3_, O-K, Mn-L_2,3_ and Fe-L_2,3_ edges.

Valence state mapping was performed using multiple linear least squares (MLLS) fitting of the region covering the L_3_ and L_2_ white lines for Mn using internal references from the datasets for the Mn^3+^ and Mn^4+^ near edge shapes, on the assumption that the whole film exists as some mixture of these two oxidation states (in accordance with our previous work [13]).

## Results

**Fig. 1:**
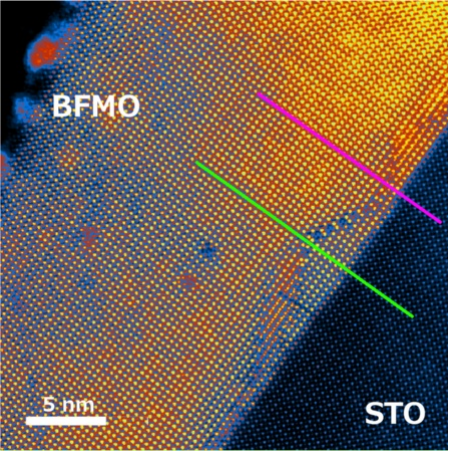
HAADF image of the BFMO thin film on SrTiO_3_. *Two lines* are shown for profiles taken

Figure 1 shows a typical HAADF STEM image of an area of the BFMO film containing antiphase boundaries. These may be recognised as the same stepped structure of antiphase boundary as has recently been reported by MacLaren et al*.* in Bi_0.85_Nd_0.15_Fe_0.9_Ti_0.1_O_3_ [18]. It is clear that these stepped structures are nucleated at or just above the interface with the SrTiO_3_ and then grow stepwise upwards until they intersect another stepped boundary nucleated elsewhere at the substrate-film interface, creating a pyramidal structure. There is also evidence from overlapped regions, such as that indicated in Fig. 1, and from focal series collected in some areas, that the overall 3D structure is of pyramids of BFMO connected in direct epitaxial relationship to the SrTiO_3_ separated from the outer part of the film by this pyramidal antiphase boundary (APB) network. Thus, the A-site positions in the outer part of the film are always in an antiphase relationship with the A-sites in the SrTiO_3_ substrate: this has been observed in multiple TEM samples and for two different film thicknesses.

**Fig. 2:**
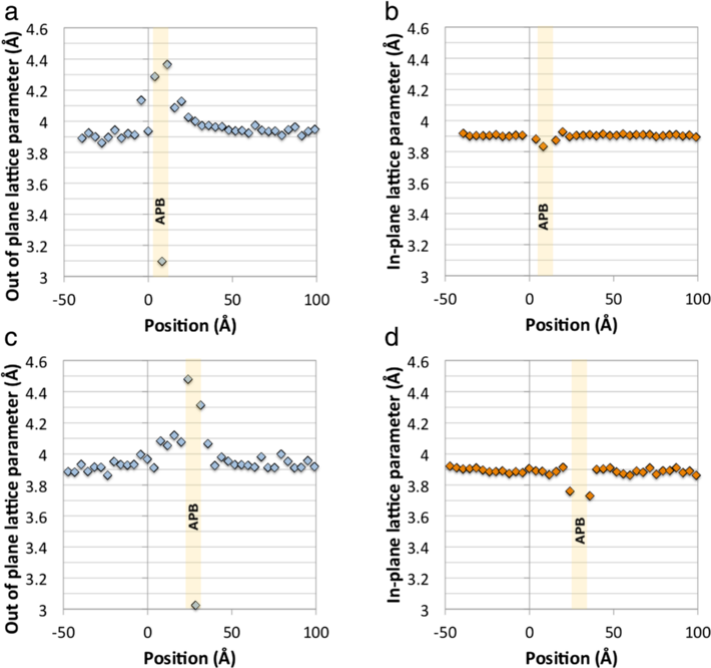
Quantification of the structure in Fig. 1. **a** Out-of-plane and **b** in-plane lattice parameters as a function of distance along the *pink line* shown in Fig. 1. **c** Out-of-plane and **d** in-plane lattice parameters as a function of distance along the *green line* shown in Fig. 1. In all cases, the position values <0 are in the SrTiO_3_ substrate, and position values >0 are in the BFMO film

Figure 2a shows a plot of the out-of-plane lattice parameter along the pink line shown in Fig. 1, which passes through the location where the APB is flat and about 1 unit cell above the STO-BFMO interface. This shows a jump to about 4.15 Å at the film-substrate interface, followed by larger jumps to either side of the APB just above the interface, with a short spacing of about 3.1 Å in between. This is entirely in accord with the previously published structure of flat terraces on such APBs [16], where the first cell on either side of the APBs has a huge *c* parameter of about 4.3–4.4 Å due to the stabilisation of a super-tetragonal, highly polar phase in response to the high charge density at the boundary [16]. Above the boundary, the out-of-plane parameter decays gradually over 5 or 6 unit cells back to an equilibrium level, which is slightly higher than that in the SrTiO_3_. Using the average spacing in the SrTiO_3_ as an internal calibration of 3.905 Å, this new level is a little higher at ~3.93 Å. For comparison, the in-plane lattice parameter is plotted in Fig. 2b along the same line. In this case, this is almost the same in the BFMO as in the STO, with just a slight disturbance by the internal structure of the APB.

Figure 2c shows a plot of the out-of-plane lattice parameter along the green line in Fig. 1. This line runs through the centre of a pyramid, and the plot is surprisingly different. Specifically, there is no lattice parameter jump at the film-substrate interface, but the whole area inside the pyramid has a large out-of-plane parameter of about 4.08 Å. There is then the usual big peak to about 4.4 Å, due to the stabilisation of a super-tetragonal polar-ordered phase by the electric field from the boundary [16]; the drop to ~3 Å; and the second peak of about 4.4 Å (as explained above), when the line passed through the APB at the tip of the pyramid. This is followed by the decay back to an equilibrium value of the lattice parameter, which in this case is ~3.94 Å. Again, for comparison, the in-plane parameter is shown for comparison in Fig. 2d. As for Fig. 2b, there is no significant change of the in-plane parameter from the STO value, either inside or outside the pyramid, except the minor disturbances at the APB itself.

All this provides an interesting counterpart to previously published work where crystallographic analysis was mainly performed using X-ray diffraction [13], providing highly accurate average values of film and substrate lattice parameters, but little idea of the detailed variation of the crystal parameters within the film. The present work clearly supports the conclusions of our previous publication that the films are highly coherent with the substrate and that the in-plane parameters are constrained to those of the STO. However, it is now clear that the out-of-plane parameter shows locally significant variations within the film. Those areas that are in direct epitaxial contact with the substrate show a significantly enhanced *c*:*a* ratio with *c* values close to 4.1 Å. This is significantly above the total *c* parameter for the films from X-ray diffraction of about 4.015 Å [13]. This is compensated for by the fact that outside the APB, the *c* parameter decays back to something not far from the expected bulk value. This range of *c* parameters will give an average for the whole film not far from 4 Å and a rather spread reciprocal space peak for the film, as observed in Fig. 1c of Choi et al*.* [13].

**Fig. 3:**
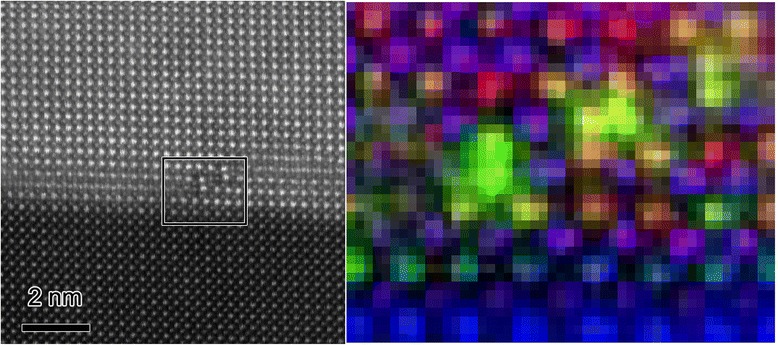
Nucleation of an antiphase boundary at the SrTiO_3_:BFMO interface. **(left)** HAADF image showing antiphase boundary features either at (*left*) or above (*right*) the film-substrate interface, as well as showing the area used for EELS-SI. **(right)** Elemental map from processing of the EELS-SI data where Fe is *red*, Mn is *green*, Ti is *blue* and the simultaneously acquired HAADF signal is *purple*

Figure 3 shows an atomic resolution chemical map of the area where an APB is in direct contact with the underlying SrTiO_3_. It should be noted that the APB does not lie right on the interface, even on the left-hand side, but one cell inside the BFMO. Chemical maps show that there is an enrichment of Mn on the B-sites at the interface on the left-hand side. This corresponds to where the APB runs right along the film-substrate interface, and there is also a small but significant concentration of Ti along with the Mn on the B-sites at the interface, as reported previously by Choi et al*.* [13]. On the right-hand side, where the APB steps up and away from the film-substrate interface, there is also a strong enrichment of Mn to the boundaries. The fact that Mn is already segregating to the film-substrate interface and that a flat APB forms here clearly shows that Mn is associated with the nucleation of the APBs in the first unit cell above the film-substrate interface, possibly in association with a little Ti from the substrate. In our previous studies [16], it was found that these boundaries were formed in BiFeO_3_ in the presence of excess Ti^4+^, and it seems that Mn can play a similar role. It is then seen that the Mn segregation is key to how these boundaries then step away from the film-substrate interface into the pyramid structures shown in Fig. 1.

**Fig. 4:**
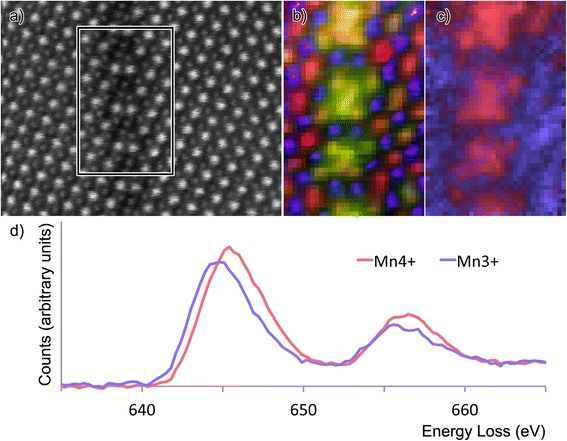
Atomic resolution chemical maps of a stepped region of an antiphase boundary. **a** Survey image. **b** Composite image of Fe (*red*), HAADF signal (*purple*) and Mn signal (*green*). **c** Composite image showing the Mn^3+^ (*lilac*) and Mn^4+^ (*pink*) MLLS fits. **d** Standard spectra used for this MLLS fit

Figure 4 shows atomic resolution chemical maps of a stepped area of the APB showing that, whilst Fe is present both in B-sites in the surrounding perovskite and in the boundary, Mn is strongly segregated to the B-sites in the boundary. It was clear from studying the EEL spectra from this spectrum image, as shown in Fig. 3d, that the Mn white lines were shifting and changing shape between the perovskite and the boundary, suggesting that the oxidation state is changing. The trends are much like those seen previously by Garvie and Craven [25], whereby the edge onset moves to higher energy with increasing oxidation state, with a concurrent decrease in the L_3_:L_2_ ratio, as later used by Wang et al. and others [26–28] for measuring Mn oxidation states. Mapping using the L_3_:L_2_ ratio was used by Choi et al*.* [13] to show a change of Mn oxidation state in these films from Mn^3+^ in the perovskite to Mn^4+^ on the interface to the SrTiO_3_. In the current work, MLLS fitting was used to treat a background-subtracted Mn edge spectrum image of this area (640–665 eV energy loss) as a linear sum of two extreme components, one from the matrix and another from the step in the boundary. The former must be Mn^3+^ in accord with Choi et al*.* [13], and the latter was believed to be close to Mn^4+^. The results are shown in Fig. 3c which clearly show that all the Mn in the boundary steps oxidises to Mn^4+^.

## Discussion

With the benefit of this atomic resolution chemical analysis, it is now possible to compare this stepped APB structure to that previously reported in Nd,Ti co-doped BiFeO_3_ [18]. In that work, flat APB sections were formed with Ti^4+^ at the core, and the steps on APBs were always formed from four closely separated columns of Fe^3+^, which are linked together as edge-sharing FeO_6_ octahedra. In this work, it is shown that very similar steps can be created in a BiFeO_3_ derivative (Bi(Fe,Mn)O_3_) using Mn^4+^ to form the steps, in preference to Fe^3+^. One reason for the segregation of Mn to these boundaries could be that the desired BiFe_0.5_Mn_0.5_O_3_ composition is less stable than a mixture of Fe-rich Bi(Fe,Mn)O_3_ and phases richer in Mn. If this were the case, there would be a clear driving force to phase separation, which when it occurs for slow film deposition rates would lead mainly to the formation of local, non-stoichiometric defects, rather than larger scale phase separation into second phases. The fact that there is very little Ti present in the film, apart from possibly a little diffused from the substrate in the first atomic layer, may explain why flat APB sections are only supported right at the interface with the substrate, seeing as previous observations found Ti was vital to the stabilisation of flat APBs [16].

There is then the question of why the Mn changes oxidation state to Mn^4+^ in the boundaries. It is well known that these boundaries contain an excess of negative charge in the core [16, 18]: this conclusion was easily reached in our previous publications by counting oxygen ions and the cations and calculating the net charge density. One way that this excess charge could be reduced, if not eliminated entirely, would be to increase the oxidation state of the cations. This, therefore, explains why Mn in the boundaries is in the higher Mn^4+^ oxidation state and may provide an additional energetic reason why Mn is preferred to Fe for the cores of the steps, since Mn readily supports higher oxidation states than Fe. Whatever is the case, the stepped boundaries are still negatively charged in the core, and this is estimated to correspond to a charge density of −0.55 C m^−2^ for the core as Mn^4+^, as compared to the previously published figure of −1.09 C m^−2^ for the core as Fe^3+^ [18]. The fact that the boundary remains charged is also attested to by the fact that the surrounding material is polarised towards the APB with the Bi atoms close to the APB not sitting in the middle of the space between four surrounding B-sites but always offset towards the APB, as also observed in our previous studies [16, 18].

Since it is shown that the strain in the film is maximised when the film is in direct epitaxial contact with the substrate, and relaxed by the presence of Mn-rich APBs, it is clear what must be done to further improve the properties of these materials. APBs are nucleated at the film-substrate interface, and this seems to be associated with the preferential segregation of Mn^4+^ to the B-sites next to the interface, which possibly occurs due to the interaction with Ti^4+^ ions diffusing into the first layer or two of the film from the substrate. It would therefore seem sensible to suggest that future developments in film growth should concentrate on the elimination of Mn diffusion to the film-substrate interface. This could, perhaps, be achieved by first depositing a few atomic layers of pure BiFeO_3_ before turning the deposition to BiFe_0.5_Mn_0.5_O_3_ to prevent any interaction of Mn and Ti.

## Conclusions

In conclusion, using atomic-resolution structural and spectroscopic characterisation we have found that the growth of bismuth ferrite manganite of target composition BiFe_0.5_Mn_0.5_O_3_ on SrTiO_3_ results in the formation of Mn^4+^-rich APBs emanating from the film-substrate interface. These then form a stepped structure and intersect with one another to form a structure of approximate pyramids of film in direct 1:1 epitaxial relationship to the substrate. The effect of the pyramidal APBs is to leave the outer layer of the film in an antiphase relationship to the substrate. This has the effect of concentrating strain close to the interface but relaxing it in the outer layers of the film. The formation of the APBs appears to be driven by a tendency towards phase separation, namely into Mn-rich and Mn-poor phases.

Finally, more broadly, the finding of this work highlights the challenges involved in depositing homogeneous complex oxide thin films on the limited number of mostly lattice mismatched single-crystal substrates which are available and presents the case for use of suitable buffer layers to prevent deleterious chemical interactions in film growth.

## References

[1] Sosnowska I, Peterlinneumaier T, Steichele E (1982). Spiral magnetic-ordering in bismuth ferrite. J Physics C-Solid State Phys.

[2] Teague JR, Gerson R, James WJ (1970). Dielectric hysteresis in single crystal BiFeO3. Solid State Commun.

[3] Lebeugle D, Colson D, Forget A, Viret M, Bataille AM, Gukasov A (2008). Electric-field-induced spin flop in BiFeO3 single crystals at room temperature. Phys Rev Lett.

[4] Catalan G, Scott JF (2009). Physics and applications of bismuth ferrite. Adv Mater.

[5] Yang SY, Martin LW, Byrnes SJ, Conry TE, Basu SR, Paran D, Reichertz L, Ihlefeld J, Adamo C, Melville A, Chu YH, Yang CH, Musfeldt JL, Schlom DG, Ager JW, Ramesh R (2009). Photovoltaic effects in BiFeO3. Appl Phys Lett.

[6] Xu XS, Ihlefeld JF, Lee JH, Ezekoye OK, Vlahos E, Ramesh R, Gopalan V, Pan XQ, Schlom DG, Musfeldt JL (2010). Tunable band gap in Bi(Fe1-xMnx)O3 films. Appl Phys Lett.

[7] Fujino S, Murakami M, Anbusathaiah V, Lim SH, Nagarajan V, Fennie CJ, Wuttig M, Salamanca-Riba L, Takeuchi I (2008). Combinatorial discovery of a lead-free morphotropic phase boundary in a thin-film piezoelectric perovskite. Appl Phys Lett.

[8] Karimi S, Reaney IM, Levin I, Sterianou I (2009). Nd-doped BiFeO3 ceramics with antipolar order. Appl Phys Lett.

[9] Karimi S, Reaney IM, Han Y, Pokorny J, Sterianou I (2009). Crystal chemistry and domain structure of rare-earth doped BiFeO3 ceramics. J Mater Sci.

[10] Kan D, Palova L, Anbusathaiah V, Cheng CJ, Fujino S, Nagarajan V, Rabe KM, Takeuchi I (2010). Universal behavior and electric-field-induced structural transition in rare-earth-substituted BiFeO3. Adv Func Mater.

[11] Choi EM, Patnaik S, Weal E, Sahonta SL, Mecklenburg G, Wang H, Bi Z, Xiong J, Blamire MG, Jia QX, MacManus-Driscoll JL (2011). Strong room temperature magnetism in highly resistive strained thin films of BiFe0.5Mn0.5O3. Appl Phys Lett.

[12] Zeches RJ, Rossell MD, Zhang JX, Hatt AJ, He Q, Yang CH, Kumar A, Wang CH, Melville A, Adamo C, Sheng G, Chu YH, Ihlefeld JF, Erni R, Ederer C, Gopalan V, Chen LQ, Schlom DG, Spaldin NA, Martin LW, Ramesh R (2009). A strain-driven morphotropic phase boundary in BiFeO3. Science.

[13] Choi EM, Fix T, Kursumovic A, Kinane CJ, Arena D, Sahonta SL, Bi ZX, Xiong J, Yan L, Lee JS, Wang HY, Langridge S, Kim YM, Borisevich AY, MacLaren I, Ramasse QM, Blamire MG, Jia QX, MacManus-Driscoll JL (2014). Room temperature ferrimagnetism and ferroelectricity in strained, thin films of BiFe0.5Mn0.5O3. Adv Func Mater.

[14] Schaffer B, Grogger W, Kothleitner G (2004). Automated Spatial Drift Correction for EFTEM Image Series. Ultramicroscopy.

[15] MacLaren I, Villaurrutia R, Schaffer B, Houben L, Pelaiz-Barranco A (2012). Atomic-scale imaging and quantification of electrical polarisation in incommensurate antiferroelectric lanthanum-doped lead zirconate titanate.. Adv Func Mater.

[16] MacLaren I, Wang LQ, Morris O, Craven AJ, Stamps RL, Schaffer B, Ramasse QM, Miao S, Kalantari K, Sterianou I, Reaney IM (2013). Local stabilisation of polar order at charged antiphase boundaries in antiferroelectric (Bi0.85Nd0.15) (Ti0.1Fe0.9) O3. APL Materials.

[17] MacLaren I, Wang LQ, Schaffer B, Ramasse QM, Craven AJ, Selbach SM, Spaldin NA, Miao S, Kalantari K, Reaney IM (2013). Novel nanorod precipitate formation in neodymium and titanium codoped bismuth ferrite. Adv Func Mater.

[18] MacLaren I, Wang L, Craven AJ, Ramasse QM, Schaffer B, Kalantari K, Reaney IM (2014). The atomic structure and chemistry of Fe-rich steps on antiphase boundaries in ti-doped Bi0.9Nd0.15FeO3. APL Materials.

[19] Lucas G, Burdet P, Cantoni M, Hebert C (2013). Multivariate statistical analysis as a tool for the segmentation of 3D spectral data. Micron.

[20] Leapman RD, Swyt CR (1988). Separation of overlapping core edges in electron-energy loss spectra by multiple-least-squares fitting. Ultramicroscopy.

[21] Manoubi T, Tence M, Walls MG, Colliex C (1990). Curve fitting methods for quantitative-analysis in electron-energy loss spectroscopy. Microsc Microanal Microstruct.

[22] Verbeeck J, Van Aert S, Bertoni G (2006). Model-based quantification of EELS spectra: including the fine structure. Ultramicroscopy.

[23] Verbeeck J, Van Aert S (2004). Model based quantification of EELS spectra. Ultramicroscopy.

[24] Thomas PJ, Twesten RD (2012). A simple, model based approach for robust quantification of EELS spectra and spectrum-images. Microsc Microanal.

[25] Garvie LAJ, Craven AJ (1994). High-resolution parallel electron-energy-loss spectroscopy of Mn L2,3-edges in inorganic manganese compounds. Phys Chem Miner.

[26] Wang ZL, Yin JS, Mo WD, Zhang ZJ, Phys J (1997). In-situ analysis of valence conversion in transition metal oxides using electron energy-loss spectroscopy. Chem B.

[27] Wang ZL, Yin JS, Jiang YD (2000). EELS analysis of cation valence states and oxygen vacancies in magnetic oxides. Micron.

[28] Wang YQ, Maclaren I, Duan XF (2001). EELS analysis of manganese valence states in rare-earth manganites (La1-xYx) 0.5 (Ca1-ySry) 0.5 MnO3. Mat Sci Eng a-Struct.

